# Prognostic effects of different treatment modalities for hypopharyngeal squamous cell carcinoma: Experience of two tertiary hospitals in Southwestern China^☆^

**DOI:** 10.1016/j.heliyon.2024.e28496

**Published:** 2024-03-26

**Authors:** Junhong Li, Shanshan He, Jifeng Liu, Di Deng, Yijun Dong, Wendu Pang, Mao Minzi, Ke Qiu, Jing Zeng, Yao Song, Yufang Rao, Yu Zhao, Xinyin Xu, Shichuan Zhang, Ren Jianjun

**Affiliations:** a Department of Otolaryngology-Head & Neck Surgery and National Clinical Research Center for Geriatrics, West China Hospital, Sichuan University, Chengdu, China; b Department of Radiation Oncology, Sichuan Cancer Hospital & Institute, School of Medicine, University of Electronic Science and Technology of China, Sichuan Cancer Center, Radiation Oncology Key Laboratory of Sichuan Province, Chengdu, China; c Sichuan Provincial People's Hospital Jinniu Hospital, China; d Sichuan Center for Disease Control and Prevention, Chengdu, China

**Keywords:** Hypopharyngeal squamous cell carcinoma, Curative treatment, Surgery-based multimodality treatment, Chemoradiotherapy, Overall survival

## Abstract

**Background:**

The prognostic effects of different treatment modalities on patients with hypopharyngeal squamous cell carcinoma (HPSCC) remain unclear.

**Methods:**

HPSCC patients diagnosed and treated at either West China Hospital or Sichuan Cancer Hospital between January 1, 2009, and December 31, 2019, were enrolled in this retrospective, real-world study. Survival rates were presented using Kaplan–Meier curves and compared using log-rank tests. Univariable and multivariable Cox proportional hazards regression models were used to identify the predictors of overall survival (OS). Subgroup analyses were conducted for patients with advanced-stage HPSCC (stages III and IV and category T4).

**Results:**

A total of 527 patients with HPSCC were included. Patients receiving SRC (surgery, radiotherapy [RT], and chemotherapy) showed the best OS (p < 0.0001). In comparison with RT alone, both surgery alone (all cases: hazard ratio [HR] = 0.39, p = 0.0018; stage IV cases: HR = 0.38, p = 0.0085) and surgery-based multimodality treatment (SBMT; all cases: HR = 0.27, p < 0.0001; stage IV cases: HR = 0.30, p = 0.00025) showed prognostic benefits, while SBMT also showed survival priority over chemoradiotherapy (CRT; all cases: HR = 0.52, p < 0.0001; stage IV cases: HR = 0.59, p = 0.0033). Moreover, patients who underwent surgery alone had comparable OS to those who underwent SBMT (all patients: p = 0.13; stage IV cases: p = 0.34), while CRT yielded similar prognostic outcomes as RT alone (all patients: p = 0.054; stage IV cases: p = 0.11).

**Conclusions:**

Surgery alone was comparable to SBMT and superior to RT/CRT in terms of OS in patients with HPSCC. We suggest that surgery should be encouraged for the treatment of HPSCC, even in patients with advanced-stage disease.

## Highlights

• HPSCC patient management experience from two large hospital.
• Surgery alone has similar survival benefit as surgery-based multimodality treatment.
• Surgery-based treatments were superior over radiotherapy and/or chemotherapy.
• Surgery was recommended even for advanced HPSCC patients.

## 1 Introduction

Hypopharyngeal squamous cell carcinoma (HPSCC) is an uncommon head and neck cancer, constituting 0.4% of all newly diagnosed cancers [1], and showing an increasing incidence over the past decades [2]. Although the prognosis of HPSCC has improved marginally, the 5-year survival rate of HPSCC patients remains approximately 30%–35% [[3], [4], [5]]. Possible reasons for poor prognosis include advanced stage at diagnosis, multifocal nature, and early submucosal spread [3].

For patients with early-stage HPSCC (T1N0 and some T2N0 cases), both the National Comprehensive Cancer Network (NCCN) [6] and Chinese Society of Clinical Oncology (CSCO) [7] guidelines recommend radiotherapy (RT) alone or surgery alone (or followed by adjuvant RT or chemoradiotherapy [CRT] when necessary) as curative options. For patients with locally advanced disease, four sets of curative treatments are recommended: (1) surgery alone (not for T4 patients) or followed by adjuvant RT/CRT; (2) induction chemotherapy (IC) followed by surgery/RT/CRT; (3) concurrent CRT; and (4) RT alone (only in the CSCO guidelines). RT-based nonsurgical approaches can largely preserve laryngeal function and are therefore widely applied in Western countries [3,8]. However, nonsurgical approaches in HPSCC may result in higher rates of locoregional recurrence [9], and salvage laryngopharyngectomy, which is characterized by extensive surgical scopes and high complication rates [10,11], is often the only option for cases where these nonsurgical treatments have failed, including cases showing an inadequate response following IC, residual disease after CRT, or long-term recurrence [5,9]. Consequently, the preferred treatment modality for HPSCC remains controversial [5,12,13], and a more comprehensive understanding of the prognostic effects of different treatments in HPSCC patients is needed.

In this 10-year real-world study from two tertiary hospitals in southwestern China, we aimed to explore the effects of different treatment modalities on survival rates in patients with HPSCC and to compare the prognostic effects of surgery- and RT-based treatments.

## 2 Materials and methods

### 2.1 Data sources and participants

This retrospective real-world observational study was conducted as a joint effort of two tertiary hospitals and approved by the Ethics Committee on Biomedical Research, West China Hospital of Sichuan University (WCH No. 2019–357, issued on July 18, 2019) and Ethics Committee for Medical Research and New Medical Technology of Sichuan Cancer Hospital (SCH, KY-2022-058, issued on March 16, 2022). WCH is the largest tertiary hospital in southwestern China and provides high-quality medical services to more than 200 million people (http://www.stats.gov.cn/tjsj/zxfb/202105/t20210510_1817179.html), while SCH is the largest cancer-specific hospital in Sichuan Province. These two hospitals together cover most HPSCC patients in Sichuan Province. Patients with primary HPSCC diagnosed and treated at either WCH or SCH between January 1, 2009 and December 31, 2019 were retrospectively and consecutively enrolled in this real-world study. Patients were excluded if (1) they were not treated at either of the two hospitals, (2) the treatment information was incomplete, (3) no information was available on the American Joint Committee on Cancer TNM stage (8th edition), or (4) no survival information was available. Ultimately, 527 patients were included in the subsequent analyses (Supplementary Figure 1).

### 2.2 Measurements

We collected data on demographic (age at diagnosis, sex, marital status, body mass index [BMI]), lifestyle (smoking status, alcohol consumption frequency), clinicopathological characteristics (site of primary tumor, laterality of primary tumor, differential grade, histology, and TNM stage), and treatment-related information (surgery, RT, and chemotherapy) from electronic inpatient records and pathological reports.

### 2.3 Treatment regimens

In the two hospitals, typical operations performed for patients with HPSCC included total/partial laryngopharyngectomy/pharyngectomy or resection of the primary tumor, unilateral or bilateral neck dissection, total/hemi/no thyroidectomy, and flap reconstruction. The exact operation strategies mainly depended on the involved primary tumor site and structures, TNM stage, experience level of the surgeons, and patients’ physical conditions and willingness. For RT, the typical regimen involved a total dose of 66–72 Gy over 30–33 fractions. In addition, the commonly used chemotherapy regimens were (1) docetaxel, cisplatin, and 5-fluorouracil (TPF), (2) paclitaxel and cisplatin (TP), and (3) docetaxel and cisplatin (DP). The regimens for concurrent CRT were usually (1) cisplatin or (2) paclitaxel albumin.

Since chemotherapy alone is not recommended as a curative approach by the guidelines, we first categorized the treatment modalities into noncurative (chemotherapy alone) and curative (all other treatment options) treatments. Among the curative treatments, surgery plus chemotherapy (SC), surgery plus RT (SR), and surgery plus CRT (SRC) were defined as surgery-based multimodality treatment (SBMT).

### 2.4 Follow-up and outcomes

All patients were followed up with regular outpatient visits and telephone assessments. Survival status and date of death were mainly based on telephone assessments and the Death Cause Registration Reporting System of Sichuan Province from the Sichuan Center for Disease Control and Prevention. Overall survival (OS) was calculated from the date of diagnosis to the date of death from any cause or the last follow-up (May 1st, 2020), with death considered as an event. Patients were censored if they were alive at the date of last follow-up.

### 2.5 Statistical analysis

We analyzed all patients and performed subgroup analyses for patients with stage III or IV and category T4 disease. Continuous variables were presented as means and standard deviations or medians and ranges. Categorical variables were presented as numbers and percentages. Survival rates were presented using Kaplan–Meier curves and compared using log-rank tests. Univariable Cox proportional hazards regression models were used to assess the prognostic effects of each variable on OS, and significant prognostic factors and treatments were included in multivariable Cox proportional hazards models. The adjusted factors in the multivariable Cox proportional hazards models for the full sample included age at diagnosis, frequency of alcohol consumption, laterality of the primary tumor, differential grade, TNM categories, surgery, RT, and chemotherapy.

All analyses were conducted using R software (version 4.2.0, https://www.r-project.org/). Statistical significance was set at p < 0.05.

## 3 Results

### 3.1 Baseline characteristics of HPSCC patients

A total of 527 consecutive patients with HPSCC diagnosed at WCH and/or SCH between 2009 and 2019 were enrolled (Table 1); the maximum follow-up duration was 140 months. Approximately 286 patients died at the end of follow-up, and the median survival time and 5-year OS rate in our HPSCC cohort were 37.1 months and 39.9% (95% confidence interval [CI]: 35.4%–45.0%), respectively. Most HPSCC patients were male (98%), married (99%), smokers (85%), heavy drinkers (68%), and diagnosed with stage III/IV disease (72%). Most primary cancers arose from the pyriform fossa (87%) and were unilateral (94%). Only 2% of the patients presented with distant metastases at the time of diagnosis. The most common treatment was CRT (39%), followed by SRC (20%), chemotherapy alone (13%), surgery alone (13%), SR (9%), and RT alone (4%), and the least common treatment modality was SC (3%). Similar distributions of characteristics were also observed among patients with advanced HPSCC (stage IV, stage III, or category T4 disease; Supplementary Table 1). Baseline characteristics of HPSCC patients receiving different treatments were presented in Supplementary Table 2.

**Table 1:** Demographic and clinicopathological characteristics of HPSCC patients.

Covariate		Full Sample (n = 527)		Covariate		Full Sample (n = 527)	
		Frequency	Percent			Frequency	Percent
Follow-up time	Mean (SD)	37 (30.7)		T category	T1/2	146	28
	Median (Min,Max)	25.8 (1140)			T3	142	27
Age	Mean (SD)	60.2 (9.3)			T4	237	45
	Median (Min,Max)	60 (35,90)			Missing	N = 2	
Sex	Male	514	98	N category	N0	106	20
	Female	13	2		N1	96	18
Marital status	Married	521	99		N2	252	48
	Unmarried	6	1		N3	69	13
BMI	Normal	309	65		Missing	N = 4	
	Underweight	67	14	M category	M0	512	98
	Overweight/obesity	97	21		M1	13	2
	Missing	N = 54			Missing	N = 2	
Smoking status	No	76	15	Stage	I/II	27	5
	Yes	448	85		III	89	17
	Missing	N = 3			IV	410	78
Drinking frequency	Never	125	24		Missing	N = 1	
	Always	359	68	Surgery	No	295	56
	Sometimes	43	8		Yes	232	44
Site of primary tumor	Pyriform fossa	441	87	Chemotherapy	No	131	25
	Postwall	46	9		Yes	396	75
	Post-cricoid	20	4	Radiotherapy	No	153	29
	Missing	N = 20			Yes	374	71
Laterality of primary tumor	Unilateral	363	94	Combined treatment modality	SRC	103	20
	Both	25	6		Chemotherapy alone	69	13
	Missing	N = 139			RT alone	20	4
Differential grade	Low	201	60		CRT	206	39
	Medium	94	28		Surgery alone	66	13
	High	39	12		SC	18	3
	Missing	N = 193			SR	45	9

HPSCC: hypopharyngeal squamous cell carcinoma; SD: standard deviation; BMI: body mass index; SRC: surgery + radiotherapy + chemotherapy; RT: radiotherapy; CRT: chemoradiotherapy; SC: surgery + chemotherapy; SR: surgery + radiotherapy.

### 3.2 Prognostic factors for HPSCC

Univariable Cox proportional hazards regression models were used to identify the prognostic factors for HPSCC. The results suggested that age at diagnosis, frequency of alcohol consumption, laterality of the primary tumor, differential grade, T category, N category, TNM stage, and treatment were significant predictors of OS (Supplementary Tables 3–4). However, in our multivariable Cox proportional hazards models, only differential grade, N category, and surgery remained significant (Table 2). In the subgroup analysis of patients with advanced HPSCC (stage IV, stage III, and category T4), only surgery was a consistently significant predictor of OS (Supplementary Table 5).

**Table 2:** Multivariable Cox proportional hazards regression models identified some predictors of HPSCC overall survival.

Covariate		HR(95%CI)	p-value	Global p-value
Age	Age	1.01 (0.99,1.04)		0.2
Drinking frequency	Never	Reference		0.3
	Always	1.40 (0.83,2.37)	0.2	
	Sometimes	1.69 (0.82,3.50)	0.15	
Laterality of primary tumor	Unilateral	Reference		0.051
	Both	1.95 (1.00,3.79)		
Differential grade	Low	Reference		**0.017**
	High	2.36 (1.30,4.28)	**0.005**	
	Medium	0.86 (0.52,1.42)	0.55	
T category	T1/2	Reference		0.3
	T3	1.25 (0.73,2.12)	0.42	
	T4	1.47 (0.90,2.41)	0.13	
N category	N0	Reference		**0.005**
	N1	1.95 (0.96,4.00)	0.067	
	N2	2.87 (1.52,5.41)	**0.001**	
	N3	2.47 (1.04,5.90)	**0.042**	
M category	M0	Reference		0.97
	M1	1.03 (0.27,3.93)		
Surgery	No	Reference		**<0.001**
	Yes	0.41 (0.26,0.65)		
Radiation	No	Reference		0.81
	Yes	1.06 (0.67,1.66)		
Chemotherapy	No	Reference		**0.009**
	Yes	0.49 (0.29,0.84)		

HPSCC: hypopharyngeal squamous cell carcinoma; HR: hazard ratio; CI: confidence interval.

### 3.3 Survival of patients receiving different treatments

**Fig. 1:**
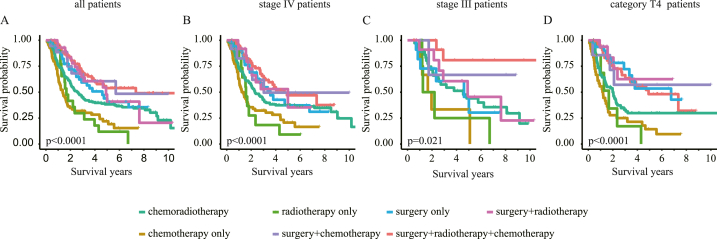
**Different treatment modalities showed distinct overall survival benefits on HPSCC patients.** Analyses were conducted in (A) all patients, (B) stage IV patients, (C) stage III patients, and (D)category T4 patients, respectively.

The OS of patients receiving different treatment modalities was distinct, and those treated with SRC showed the best survival performance (Fig. 1A–D and Supplementary Tables 2–3). We further compared the OS of patients who received different treatments from the following perspectives.

#### 3.3.1 Curative vs. noncurative treatment: curative treatments should Be encouraged even for those with advanced stages

**Fig. 2:**
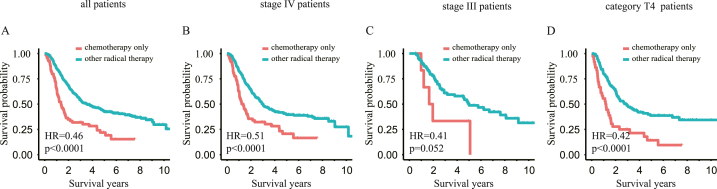
**Comparison of overall survival between HPSCC patients treated with noncurative therapy (chemotherapy only) and those treated with other curative therapies suggested that curative therapies should be recommended.** Analyses were conducted in (A) all patients, (B) stage IV patients, (C) stage III patients, and (D)category T4 patients, respectively.

Patients who received noncurative treatment (chemotherapy alone) had a significantly lower OS than those who received curative treatments (hazard ratio [HR] = 0.46, CI: 0.34–0.62, p < 0.0001; Fig. 2A). Similar results were also observed in patients with advanced HPSCC (stage IV: HR = 0.51, CI: 0.37–0.7, p < 0.0001, Fig. 2B; stage III: HR = 0.41, CI: 0.16–1.04, p = 0.052, Fig. 2C; category T4: HR = 0.42, CI: 0.28–0.62, p < 0.0001, Fig. 2D). These results suggested that curative treatment should be encouraged whenever possible, even in patients with advanced-stage HPSCC.

#### 3.3.2 RT alone vs. surgery Alone/SBMT: surgery Outperformed RT

In comparison with RT alone, both surgery alone and SBMT showed significant survival benefits (surgery alone: HR = 0.39, CI: 0.21–0.72, p = 0.0018; SBMT: HR = 0.27, CI: 0.16–0.48, p < 0.001; Supplementary Figure 2). Similar results were also observed in stage IV (surgery alone: HR = 0.38, CI: 0.18–0.8, p = 0.0085; SBMT: HR = 0.30, CI: 0.15–0.59, p = 0.0025) and category T4 patients (surgery alone: HR = 0.24, CI: 0.08–0.66, p = 0.003; SBMT: HR = 0.24, CI: 0.1–0.59, p = 0.00071; Supplementary Figure 2). When comparing different types of SBMT with RT alone, similar prognostic benefits over RT alone were observed for SC (all patients: HR = 0.31; CI: 0.13–0.76, p = 0.0071), SR (all patients: HR = 0.30, CI: 0.15–0.61, p = 0.00041; stage IV disease: HR = 0.34, CI: 0.14–0.85, p = 0.016; category T4: HR = 0.22, CI: 0.06–0.81, p = 0.014), and SRC (all patients: HR = 0.26, CI: 0.14–0.48, p < 0.0001; stage IV: HR = 0.28, CI: 0.14–0.58, p = 0.00027; category T4: HR = 0.24, CI: 0.09–0.62, p = 0.0014; Supplementary Figure 3).

#### 3.3.3 CRT vs. surgery Alone/SBMT: surgery outperformed CRT

**Fig. 3:**
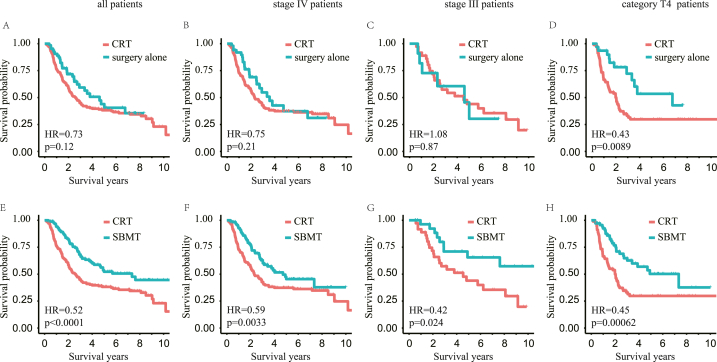
**Comparison of overall survival between HPSCC patients treated with CRT and surgery/SBMT highlighted the effects of surgery.** A–D: Survival of HPSCC patients receiving CRT and surgery alone in (A) all, (B) stage IV, (C) stage III, and (D) category T4 patients, respectively. E–H: Survival of HPSCC patients receiving CRT and SBMT in (E) all, (F) stage IV, (G) stage III, and (H) category T4 patients, respectively.

We observed that surgery alone was associated with an improved OS when compared with CRT in patients with T4 disease (HR = 0.43, CI: 0.23–0.83, p = 0.0089, Fig. 3A–D), and SBMT presented survival benefits over CRT in all subgroups (all patients: HR = 0.52, CI: 0.38–0.71, p < 0.0001, Fig. 3E; stage IV: HR = 0.59, CI: 0.42–0.84, p = 0.0033, Fig. 3F; stage III: HR = 0.42, CI: 0.19–0.91, p = 0.024, Fig. 3G; category T4: HR = 0.45, CI: 0.29–0.72, p = 0.00062, Fig. 3H). Patients treated with SR (all patients: HR = 0.59, CI: 0.35–0.97, p = 0.036; category T4: HR = 0.32, CI: 0.12–0.88, p = 0.019) or SRC (all patients: HR = 0.49, CI: 0.33–0.71, p = 0.00013; stage IV: HR = 0.58, CI: 0.39–0.87, p = 0.0072; stage III: HR = 0.18, CI: 0.04–0.77, p = 0.0092; category T4: HR = 0.49, CI: 0.29–0.82, p = 0.0057; Supplementary Figure 4) showed better OS than those treated with CRT. However, no significant prognostic difference was observed between patients treated with CRT and those treated with SC (Supplementary Figure 4).

#### 3.3.4 Surgery alone vs. SBMT and RT vs. CRT: One treatment seemed enough

We failed to observed any significant prognostic differences between surgery alone and SBMT (Supplementary Figure 5). When considering each type of SBMT, only SRC showed survival benefits over surgery alone in patients with stage III disease (HR = 0.19, CI: 0.04–0.96, p = 0.026; Supplementary Figure 6). For RT, no significant difference in OS was observed between patients receiving RT alone and those receiving CRT (Supplementary Figure 5).

## 4 Discussion

In this large-scale, retrospective, real-world observational study of HPSCC patients, we conducted a comprehensive prognostic comparison of HPSCC patients treated with different modalities and observed that patients receiving noncurative treatment showed significantly inferior OS. In addition, surgery alone showed prognostic advantages over RT alone, and SBMT was superior to both RT and CRT. We also observed that patients who underwent surgery alone presented comparable OS to patients who underwent SBMT. Similar results were observed in HPSCC patients with advanced-stage disease (stages IV and III and category T4).

We observed that HPSCC patients receiving curative treatment presented significantly better OS than those receiving noncurative treatment. Previous studies have also suggested that the OS of HPSCC patients who received curative treatment was 43% [14], while patients who received noncurative treatment or no treatment died of cancer within a short time (less than 20% of the patients could survive for more than 1 year) [14,15]. Therefore, curative treatment should be encouraged whenever possible.

The results of studies comparing the prognostic effects of surgery- and RT-based treatments are controversial. The role of IC followed by RT in advanced HPSCC patients was established by a prospective randomized phase III study conducted by the European Organization for Research and Treatment of Cancer (EORTC) in 1996 [16], and concurrent CRT was extrapolated to HPSCC after verification in laryngeal cancer [17]. These RT-based treatments aim to provide better functional preservation of organ functions, such as vocal communication, while achieving similar oncologic prognostic outcomes [[18], [19], [20], [21], [22]]. However, several subsequent studies have suggested that both late [[23], [24], [25]] and early HPSCC patients who received surgery plus adjuvant RT/CRT have superior OS and disease-specific survival than those receiving primary CRT [9,26]. In our cohort, surgery alone showed significant prognostic superiority over RT alone, and SBMT yielded more prognostic benefits than RT alone and CRT. Therefore, we concluded that surgery/SBMT still overrides RT/CRT as a curative approach for patients with HPSCC. Moreover, our study failed to observe any significant difference in the prognosis between surgery alone and SBMT in patients with advanced HPSCC (stages IV and III and category T4). These results suggested that surgery is still the mainstay for the treatment of HPSCC, and should be encouraged even in patients with advanced-stage HPSCC. Further studies are needed to verify the better prognosis we observed for surgery and to develop more effective chemotherapy and RT regimens.

Besides, we failed to observe a significant superior OS of patients receiving CRT over those receiving RT alone, which might be due to the limited number of patients receiving RT alone. And we also failed to observe significant OS benefits of SBMT over surgery alone, which might be due to unmeasured confounders and selection bias in patients receiving surgery alone and SBMT. Further studies, especially large-scale prospective cohort studies and randomized clinical trials, are needed to explore the potential benefits of CRT and SBMT.

The strengths of the current study were mainly its large HPSCC cohort, which is a rare cancer. Besides, this study was a real-world study, which was equipped with consecutive enrollment of HPSCC patients and provided the observations in real clinical practice. Furthermore, we performed comprehensive OS comparisons between HPSCC patients receiving different treatments, and subgroup analysis stratified by cancer stages were addressed, allowing a more comprehensive understanding of the prognostic effects of different treatments.

Although our retrospective real-world study enrolled a large number of patients with HPSCC and provided multiple prognostic comparisons among patients with HPSCC receiving different treatments in two large tertiary hospitals, it had some limitations. First, heterogeneous RT and/or chemotherapy regimens were used, which limited our ability to compare the prognostic value of different RT and chemotherapy regimens. Second, information on immunotherapy was limited, which made it impossible to evaluate the effects of immunotherapy. However, only a few participants underwent immunotherapy before 2019 at the two tertiary hospitals; therefore, our primary results were not biased. Third, as this study was a retrospective observational study based on electronic medical records, we had no pre-treatment overall health condition information of the patients. Besides, they were not randomized to different treatments. These limitations might lead to selection bias. For example, patients receiving surgery might be at superior overall health conditions and were tolerable to surgery, which might contribute to their superior prognosis. Indeed, when we compared the baseline characteristics of HPSCC patients receiving different treatments, some differences concerning age at diagnosis, differential grade, N stage and overall TNM stage were observed. However, patients undergoing surgery were at even older age, and subgroup analysis stratified by stage were performed to address these issues. Further large-scale prospective cohort studies and clinical randomized trials are needed to provide a more comprehensive evaluation of our observations. Fourth, since information regarding recurrence was limited, we were unable to assess the effects of different treatment modalities on the recurrence rate.

## 5 Conclusions

In conclusion, our study described the management experience for HPSCC patients in two large tertiary hospitals in southwestern China. On the basis of our findings, we suggest that SBMT, especially surgery, should be encouraged for the treatment of HPSCC, even in patients with advanced-stage disease.

## Grant supports

This work was supported by 10.13039/501100013365West China Hospital, Sichuan University [grant number ZYJC21027], National Natural Youth Science Foundation of China [grant number 82002868], 10.13039/100014717National Natural Science Foundation of China [grant number 82272777], and Sichuan Medical and Health Care Promotion Institute [grant number KY2022QN0270].

## Role of the funding source

The funders had no role in considering the study design or in the collection, analysis, interpretation of data, writing of the report, or decision to submit the article for publication. The authors declare that there is no conflict of interest.

## Ethical approval

Written informed consents were obtained from all participants and ethical approval was obtained from Ethics Committee on Biomedical Research, West China Hospital, Sichuan University (No. 2019.357, issued on July 18, 2019) and Ethics Committee for Medical Research and New Medical Technology of Sichuan Cancer Hospital (KY-2022-058, issued on March 16, 2022).

## Data availability statement

The data associated with the study has not been deposited into a publicly available repository, because the authors do not have permission to share data according to the institution's policies and ethical requirements.

## CRediT authorship contribution statement

**Junhong Li:** Writing – review & editing, Writing – original draft, Investigation, Formal analysis, Conceptualization. **Shanshan He:** Writing – review & editing, Writing – original draft, Investigation, Conceptualization. **Jifeng Liu:** Writing – review & editing, Writing – original draft, Methodology, Investigation, Conceptualization. **Di Deng:** Writing – review & editing, Writing – original draft, Formal analysis, Data curation, Conceptualization. **Yijun Dong:** Writing – review & editing, Writing – original draft, Formal analysis, Data curation, Conceptualization. **Wendu Pang:** Writing – review & editing, Writing – original draft, Formal analysis, Data curation, Conceptualization. **Mao Minzi:** Writing – review & editing, Writing – original draft, Formal analysis, Data curation, Conceptualization. **Ke Qiu:** Writing – review & editing, Writing – original draft, Validation, Formal analysis, Data curation, Conceptualization. **Jing Zeng:** Writing – review & editing, Data curation, Conceptualization. **Yao Song:** Writing – review & editing, Writing – original draft, Visualization, Validation, Formal analysis, Data curation, Conceptualization. **Yufang Rao:** Writing – review & editing, Writing – original draft, Investigation, Formal analysis, Conceptualization. **Yu Zhao:** Writing – review & editing, Supervision, Resources, Project administration, Funding acquisition, Conceptualization. **Xinyin Xu:** Writing – review & editing, Supervision, Resources, Project administration, Data curation, Conceptualization. **Shichuan Zhang:** Writing – review & editing, Writing – original draft, Supervision, Resources, Project administration, Conceptualization. **Ren Jianjun:** Writing – review & editing, Project administration, Funding acquisition, Conceptualization.

## Declaration of competing interest

The authors declare that they have no known competing financial interests or personal relationships that could have appeared to influence the work reported in this paper.
