# Supplementary material for: Prognostic effects of different treatment modalities for hypopharyngeal squamous cell carcinoma: Experience of two tertiary hospitals in Southwestern China
Source: Heliyon. 2024 Mar 26;10(7):e28496. doi: 10.1016/j.heliyon.2024.e28496 (PMC11004745; doi:10.1016/j.heliyon.2024.e28496)

Table of contents

[Supplementary Table 1: Demographic and clinicopathological characteristics of HPSCC patients with stage IV and III and category T4 disease 2](#_Toc158624946)

[Supplementary Table 2: Demographic and clinicopathological Characteristics of HPSCC patients receiving different treatments 4](#_Toc158624947)

[Supplementary Table 3: Univariable Cox proportional hazards regression analysis evaluating the predictors of overall survival in patients with HPSCC 7](#_Toc158624948)

[Supplementary Table 4: Univariable Cox proportional hazards regression analysis evaluating predictors of overall survival in stage IV, stage III, and category T4 HPSCC 8](#_Toc158624949)

[Supplementary Table 5: Multivariable Cox proportional hazards regression analysis evaluating the predictors of overall survival in stage IV, stage III, and category T4 HPSCC 11](#_Toc158624950)

[Supplementary Figure 1: Patient inclusion and exclusion flowchart 13](#_Toc158624951)

[Supplementary Figure 2: Comparison of overall survival between HPSCC patients treated with RT alone and those treated with surgery/SBMT 14](#_Toc158624952)

[Supplementary Figure 3: Comparison of overall survival between HPSCC patients treated with RT alone and those treated with different types of SBMT 15](#_Toc158624953)

[Supplementary Figure 4: Comparison of overall survival between HPSCC patients treated with CRT and those treated with different types of SBMT 16](#_Toc158624954)

[Supplementary Figure 5: Comparison of overall survival between HPSCC patients treated with surgery alone and SBMT, and between HPSCC patients treated with RT alone and CRT, respectively 17](#_Toc158624955)

[Supplementary Figure 6: Comparison of overall survival between HPSCC patients treated with surgery alone and those treated with different types of SBMT 18](#_Toc158624956)

Supplementary Table 1: Demographic and clinicopathological characteristics of HPSCC patients with stage IV and III and category T4 disease

| **Covariate** | | **Stage IV (n=410)** | | **Stage III (n=89)** | | **Category T4 (n=237)** | |
| --- | --- | --- | --- | --- | --- | --- | --- |
|  |  | **Frequency** | **Percent** | **Frequency** | **Percent** | **Frequency** | **Percent** |
| **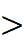Age** | **Mean (SD)** | 60 (9.5) | | 61.2 (8.7) | | 60 (9.6) |  |
|  | **Median (Min,Max)** | 60 (35,90) | | 60.2 (46,84) | | 60 (39.2,90) | |
| **Sex** | **Male** | 400 | 98 | 87 | 98 | 229 | 97 |
|  | **Female** | 10 | 2 | 2 | 2 | 8 | 3 |
| **Marital status** | **Married** | 405 | 99 | 88 | 99 | 235 | 99 |
|  | **Unmarried** | 5 | 1 | 1 | 1 | 2 | 1 |
| **BMI** | **Normal** | 237 | 64 | 56 | 75 | 133 | 61 |
|  | **Underweight** | 53 | 14 | 10 | 13 | 41 | 19 |
|  | **Overweight_obesity** | 83 | 22 | 9 | 12 | 43 | 20 |
|  | **Missing** | N=37 | | N=14 | | N=20 | |
| **Smoking status** | **No** | 60 | 15 | 12 | 13 | 34 | 14 |
|  | **Yes** | 348 | 85 | 77 | 87 | 202 | 86 |
|  | **Missing** | N=2 | | / | | N=1 | |
| **Drinking frequency** | **Never** | 93 | 23 | 25 | 28 | 57 | 24 |
|  | **Always** | 281 | 69 | 60 | 67 | 157 | 66 |
|  | **Sometimes** | 36 | 9 | 4 | 4 | 23 | 10 |
| **Site of primary tumor** | **Pyriform** | 341 | 87 | 77 | 89 | 198 | 87 |
|  | **Postwall** | 34 | 9 | 7 | 8 | 22 | 10 |
|  | **Post-cricoid** | 17 | 4 | 3 | 3 | 8 | 4 |
|  | **Missing** | N=18 | | N=2 | | N=9 | |
| **Laterality of primary tumor** | **Unilateral** | 274 | 93 | 64 | 94 | 157 | 92 |
|  | **Both** | 21 | 7 | 4 | 6 | 13 | 8 |
|  | **Missing** | N=115 | | N=21 | | N=67 | |
| **Differential grade** | **Low** | 164 | 63 | 26 | 48 | 92 | 62 |
|  | **Medium** | 67 | 26 | 20 | 37 | 40 | 27 |
|  | **High** | 28 | 11 | 8 | 15 | 17 | 11 |
|  | **Missing** | N=151 | | N=35 | | N=88 | |
| **T category** | **T1/2** | 89 | 22 | 30 | 34 | / | |
|  | **T3** | 82 | 20 | 59 | 66 |  |  |
|  | **T4** | 237 | 58 | / | |  |  |
|  | **Missing** | N=2 | | / | |  |  |
| **N category** | **N0** | 43 | 11 | 37 | 42 | 43 | 18 |
|  | **N1** | 44 | 11 | 52 | 58 | 44 | 19 |
|  | **N2** | 252 | 62 | / | | 122 | 52 |
|  | **N3** | 69 | 17 |  |  | 27 | 11 |
|  | **Missing** | N=2 | | / | | N=1 | |
| **M category** | **M0** | 395 | 97 | / | | 228 | 97 |
|  | **M1** | 13 | 3 |  |  | 7 | 3 |
|  | **Missing** | N=2 | |  |  | N=2 | |
| **Surgery** | **No** | 241 | 59 | 47 | 53 | 135 | 57 |
|  | **Yes** | 169 | 41 | 42 | 47 | 102 | 43 |
| **Radiotherapy** | **No** | 125 | 30 | 21 | 24 | 75 | 32 |
|  | **Yes** | 285 | 70 | 68 | 76 | 162 | 68 |
| **Chemotherapy** | **No** | 90 | 22 | 28 | 31 | 57 | 24 |
|  | **Yes** | 320 | 78 | 61 | 69 | 180 | 76 |
| **Follow-up time** | **Mean (SD)** | 33.7 (28.6) | | 48.4 (36.3) | | 31 (27.4) | |
|  | **Median (Min,Max)** | 23.9 (1,127.2) | | 34.9 (6.1,140) | | 22.2 (1,127.2) | |
| **Combined treatment modality** | **SRC** | 80 | 20 | 15 | 17 | 46 | 19 |
|  | **Chemotherapy alone** | 62 | 15 | 6 | 7 | 36 | 15 |
|  | **RT alone** | 13 | 3 | 5 | 6 | 8 | 3 |
|  | **CRT** | 166 | 40 | 36 | 40 | 91 | 38 |
|  | **Surgery alone** | 51 | 12 | 11 | 12 | 32 | 14 |
|  | **SC** | 12 | 3 | 4 | 4 | 7 | 3 |
|  | **SR** | 26 | 6 | 12 | 13 | 17 | 7 |

HPSCC: hypopharyngeal squamous cell carcinoma; SD: standard deviation; BMI: body mass index; SRC: surgery+ radiotherapy+ chemotherapy; RT: radiotherapy; CRT: chemoradiotherapy; SC: surgery+ chemotherapy; SR: surgery+ radiotherapy.

Supplementary Table 2: Demographic and clinicopathological Characteristics of HPSCC patients receiving different treatments

| **Covariate** | | **Full Sample (n=527)** | **SRC(n=103)** | **Chemotherapy alone (n=69)** | **RT alone (n=20)** | **CRT(n=206)** | **Surgery alone (n=66)** | **SC (n=18)** | **SR (n=45)** | **p-value** |
| --- | --- | --- | --- | --- | --- | --- | --- | --- | --- | --- |
| **Age** | **Mean (SD)** | 60.2 (9.3) | 58.1 (8.6) | 60.2 (8.9) | 69.8 (10.2) | 59.2 (8.7) | 62.9 (10.7) | 59.8 (9.3) | 61.7 (7.9) | <0.001 |
|  | **Median (Min,Max)** | 60 (35,90) | 58.1 (42.2,82) | 61 (42,79) | 70.3 (53,90) | 59 (35,80) | 63.3 (39.2,86) | 58.6 (44,79.5) | 61.2 (46.9,82) |  |
| **Sex** | **Male** | 514 (98) | 103 (100) | 66 (96) | 19 (95) | 201 (98) | 64 (97) | 17 (94) | 44 (98) | 0.21 |
|  | **Female** | 13 (2) | 0 (0) | 3 (4) | 1 (5) | 5 (2) | 2 (3) | 1 (6) | 1 (2) |  |
| **Marital status** | **Married** | 521 (99) | 102 (99) | 69 (100) | 19 (95) | 205 (100) | 63 (95) | 18 (100) | 45 (100) | 0.09 |
|  | **Unmarried** | 6 (1) | 1 (1) | 0 (0) | 1 (5) | 1 (0) | 3 (5) | 0 (0) | 0 (0) |  |
| **BMI** | **Normal** | 309 (65) | 63 (66) | 47 (76) | 12 (63) | 127 (64) | 28 (58) | 10 (67) | 22 (61) | 0.15 |
|  | **Underweight** | 67 (14) | 8 (8) | 7 (11) | 5 (26) | 33 (17) | 10 (21) | 0 (0) | 4 (11) |  |
|  | **Overweight_obesity** | 97 (21) | 24 (25) | 8 (13) | 2 (11) | 38 (19) | 10 (21) | 5 (33) | 10 (28) |  |
|  | **Missing** | 54 | 8 | 7 | 1 | 8 | 18 | 3 | 9 |  |
| **Smoking status** | **No** | 76 (15) | 15 (15) | 11 (16) | 2 (11) | 30 (15) | 11 (17) | 1 (6) | 6 (13) | 0.97 |
|  | **Yes** | 448 (85) | 88 (85) | 57 (84) | 17 (89) | 175 (85) | 55 (83) | 17 (94) | 39 (87) |  |
|  | **Missing** | 3 | 0 | 1 | 1 | 1 | 0 | 0 | 0 |  |
| **Drinking frequency** | **Never** | 125 (24) | 24 (23) | 14 (20) | 5 (25) | 48 (23) | 17 (26) | 3 (17) | 14 (31) | 0.057 |
|  | **Always** | 359 (68) | 65 (63) | 54 (78) | 15 (75) | 144 (70) | 45 (68) | 11 (61) | 25 (56) |  |
|  | **Sometimes** | 43 (8) | 14 (14) | 1 (1) | 0 (0) | 14 (7) | 4 (6) | 4 (22) | 6 (13) |  |
| **Site of primary tumor** | **Pyriform** | 441 (87) | 86 (87) | 62 (93) | 20 (100) | 166 (85) | 54 (84) | 14 (82) | 39 (89) | 0.66 |
|  | **Postwall** | 46 (9) | 11 (11) | 3 (4) | 0 (0) | 19 (10) | 8 (12) | 2 (12) | 3 (7) |  |
|  | **Post-cricoid** | 20 (4) | 2 (2) | 2 (3) | 0 (0) | 11 (6) | 2 (3) | 1 (6) | 2 (5) |  |
|  | **Missing** | 20 | 4 | 2 | 0 | 10 | 2 | 1 | 1 |  |
| **Laterality of primary tumor** | **Unilateral** | 363 (94) | 82 (93) | 37 (86) | 15 (94) | 130 (95) | 50 (93) | 11 (100) | 38 (97) | 0.47 |
|  | **Both** | 25 (6) | 6 (7) | 6 (14) | 1 (6) | 7 (5) | 4 (7) | 0 (0) | 1 (3) |  |
|  | **Missing** | 139 | 15 | 26 | 4 | 69 | 12 | 7 | 6 |  |
| **Differential grade** | **Low** | 201 (60) | 58 (60) | 20 (67) | 3 (60) | 52 (61) | 38 (63) | 11 (69) | 19 (45) | 0.013 |
|  | **Medium** | 94 (28) | 32 (33) | 5 (17) | 1 (20) | 15 (18) | 19 (32) | 3 (19) | 19 (45) |  |
|  | **High** | 39 (12) | 6 (6) | 5 (17) | 1 (20) | 18 (21) | 3 (5) | 2 (12) | 4 (10) |  |
|  | **Missing** | 193 | 7 | 39 | 15 | 121 | 6 | 2 | 3 |  |
| **T category** | **T1/2** | 146 (28) | 28 (27) | 18 (26) | 8 (40) | 67 (33) | 9 (14) | 6 (33) | 10 (22) | 0.1 |
|  | **T3** | 142 (27) | 29 (28) | 15 (22) | 4 (20) | 46 (23) | 25 (38) | 5 (28) | 18 (40) |  |
|  | **T4** | 237 (45) | 46 (45) | 36 (52) | 8 (40) | 91 (45) | 32 (48) | 7 (39) | 17 (38) |  |
|  | **Missing** | 2 | 0 | 0 | 0 | 2 | 0 | 0 | 0 |  |
| **N category** | **N0** | 106 (20) | 25 (25) | 3 (4) | 4 (21) | 30 (15) | 20 (30) | 4 (22) | 20 (45) | 0.001 |
|  | **N2** | 96 (18) | 12 (12) | 14 (20) | 4 (21) | 43 (21) | 11 (17) | 2 (11) | 10 (23) |  |
|  | **N1** | 252 (48) | 56 (55) | 35 (51) | 10 (53) | 103 (50) | 30 (45) | 7 (39) | 11 (25) |  |
|  | **N3** | 69 (13) | 9 (9) | 17 (25) | 1 (5) | 29 (14) | 5 (8) | 5 (28) | 3 (7) |  |
|  | **Missing** | 4 | 1 | 0 | 1 | 1 | 0 | 0 | 1 |  |
| **M category** | **M0** | 512 (98) | 103 (100) | 66 (96) | 19 (100) | 195 (95) | 66 (100) | 18 (100) | 45 (100) | 0.076 |
|  | **M1** | 13 (2) | 0 (0) | 3 (4) | 0 (0) | 10 (5) | 0 (0) | 0 (0) | 0 (0) |  |
|  | **Missing** | 2 | 0 | 0 | 1 | 1 | 0 | 0 | 0 |  |
| **Stage** | **I/II** | 27 (5) | 8 (8) | 1 (1) | 2 (10) | 4 (2) | 4 (6) | 2 (11) | 6 (14) | 0.0078 |
|  | **III** | 89 (17) | 15 (15) | 6 (9) | 5 (25) | 36 (17) | 11 (17) | 4 (22) | 12 (27) |  |
|  | **IV** | 410 (78) | 80 (78) | 62 (90) | 13 (65) | 166 (81) | 51 (77) | 12 (67) | 26 (59) |  |
|  | **Missing** | 1 | 0 | 0 | 0 | 0 | 0 | 0 | 1 |  |
| **Follow-up time** | **Mean (SD)** | 37 (30.7) | 42.2 (33.4) | 25.8 (22.1) | 26.4 (21.2) | 39.1 (33.1) | 35.6 (26.6) | 47 (38.5) | 35.9 (25.1) | 0.0068 |
|  | **Median (Min,Max)** | 25.8 (1,140) | 32.9 (3.7,140) | 16.4 (1,90.7) | 18.6 (4.9,80.4) | 26.3 (1.7,127.2) | 27 (4.5,100.1) | 31.1 (4.2,120) | 28.5 (5.3,123.9) |  |

HPSCC: hypopharyngeal squamous cell carcinoma; SD: standard deviation; SRC: surgery+ radiotherapy+ chemotherapy; RT: radiotherapy; CRT: chemoradiotherapy; SC: surgery+ chemotherapy; SR: surgery+ radiotherapy.

Supplementary Table 3: Univariable Cox proportional hazards regression models identified some predictors for HPSCC overall survival

| **Covariate** | | **HR (95%CI)** | **p-value** | **Global p-value** | **Covariate** | | **HR (95%CI)** | **p-value** | **Global p-value** |
| --- | --- | --- | --- | --- | --- | --- | --- | --- | --- |
| **Age** | **Age** | 1.01 (1,1.03) |  | **0.038** | **N category** | **N0** | **Reference** | | **<0.001** |
| **Sex** | **Male** | Reference | | 0.14 |  | **N1** | 1.47 (0.98,2.22) | 0.062 |  |
|  | **Female** | 0.47 (0.18,1.27) |  |  |  | **N2** | 2 (1.42,2.83) | **<0.001** |  |
| **Marital status** | **Married** | Reference | | 0.61 |  | **N3** | 1.84 (1.18,2.87) | **0.0072** |  |
|  | **Unmarried** | 0.74 (0.24,2.31) |  |  | **M category** | **M0** | Reference | | **<0.001** |
| **BMI** | **Normal** | Reference | | 0.2 |  | **M1** | 3.47 (1.89,6.35) |  |  |
|  | **Underweight** | 1.15 (0.82,1.61) | 0.43 |  | **Stage** | **I/II** | Reference | | **0.0095** |
|  | **Overweight/obesity** | 0.79 (0.57,1.1) | 0.16 |  |  | **III** | 1.48 (0.75,2.94) | 0.26 |  |
| **Smoking status** | **No** | Reference | | 0.12 |  | **IV** | 2.11 (1.12,3.98) | **0.021** |  |
|  | **Yes** | 1.34 (0.93,1.94) |  |  | **Surgery** | **No** | Reference | | **<0.001** |
| **Drinking frequency** | **Never** | Reference | | **<0.001** |  | **Yes** | 0.49 (0.38,0.63) |  |  |
|  | **Always** | 1.86 (1.36,2.55) | **<0.001** |  | **Chemotherapy** | **No** | Reference | | 0.29 |
|  | **Sometimes** | 1.64 (1,2.7) | 0.052 |  |  | **Yes** | 1.17 (0.88,1.55) |  |  |
| **Site of primary tumor** | **Pyriform fossa** | Reference | | 0.83 | **Radiotherapy** | **No** | Reference | | **0.044** |
|  | **Postwall** | 0.88 (0.57,1.38) | 0.59 |  |  | **Yes** | 0.77 (0.6,0.99) |  |  |
|  | **Post-cricoid** | 1.07 (0.6,1.92) | 0.82 |  | **Combined treatment modality** | **SRC** | Reference | | **<0.001** |
| **Laterality of primary tumor** | **Unilateral** | Reference | | **0.0067** |  | **Chemotherapy alone** | 3.59 (2.33,5.53) | **<0.001** |  |
|  | **Both** | 2 (1.21,3.3) |  |  |  | **RT alone** | 3.52 (1.94,6.38) | **<0.001** |  |
| **Differential grade** | **Low** | Reference | | **0.0018** |  | **CRT** | 2.06 (1.41,3) | **<0.001** |  |
|  | **Medium** | 0.74 (0.51,1.08) | 0.12 |  |  | **Surgery alone** | 1.49 (0.91,2.45) | 0.11 |  |
|  | **High** | 1.76 (1.17,2.64) | **0.0066** |  |  | **SC** | 1.12 (0.49,2.52) | 0.79 |  |
| **T category** | **T1/2** | Reference | | **0.0055** |  | **SR** | 1.22 (0.68,2.19) | 0.5 |  |
|  | **T3** | 1.33 (0.97,1.84) | 0.078 |  | **/** | | | | |
|  | **T4** | 1.6 (1.2,2.13) | **0.0013** |  |  |  |  |  |  |

HPSCC: hypopharyngeal squamous cell carcinoma; HR: hazard ratio; CI: confidence interval; SRC: surgery+ radiotherapy+ chemotherapy; RT: radiotherapy; CRT: chemoradiotherapy; SC: surgery+ chemotherapy; SR: surgery+ radiotherapy.

Supplementary Table 4: Univariable Cox proportional hazards regression analysis evaluating predictors of overall survival in stage IV, stage III, and category T4 HPSCC

| **Covariate** | | **Stage IV** | | | **Stage III** | | | **Category T4** | | |
| --- | --- | --- | --- | --- | --- | --- | --- | --- | --- | --- |
|  |  | **HR (95%CI)** | **p-value** | **Global p-value** | **HR (95%CI)** | **p-value** | **Global p-value** | **HR (95%CI)** | **p-value** | **Global p-value** |
| **Age** | **Age** | 1.01 (1,1.03) |  | 0.082 | 1.01 (0.97,1.05) |  | 0.61 | 1.01 (0.99,1.03) |  | 0.48 |
| **Sex** | **Male** | Reference | | 0.2 | Reference | | 0.69 | Reference | | 0.17 |
|  | **Female** | 0.47 (0.15,1.48) |  |  | 0.67 (0.09,4.85) |  |  | 0.37 (0.09,1.52) |  |  |
| **Marital status** | **Married** | Reference | | 0.46 | Reference | | 0.64 | Reference | | 0.95 |
|  | **Unmarried** | 0.59 (0.15,2.37) |  |  | 1.6 (0.22,11.7) |  |  | 1.07 (0.15,7.68) |  |  |
| **BMI** | **Normal** | Reference | | 0.15 | Reference | | 0.61 | Reference | | 0.27 |
|  | **Underweight** | 1.09 (0.75,1.59) | 0.64 |  | 1.55 (0.64,3.74) | 0.33 |  | 0.88 (0.56,1.38) | 0.58 |  |
|  | **Overweight/obesity** | 0.72 (0.5,1.04) | 0.078 |  | 1.2 (0.5,2.89) | 0.69 |  | 0.66 (0.4,1.09) | 0.11 |  |
| **Smoking status** | **No** | Reference | | 0.18 | Reference | | 0.36 | Reference | | 0.2 |
|  | **Yes** | 1.33 (0.88,2.01) |  |  | 1.54 (0.61,3.89) |  |  | 1.45 (0.82,2.58) |  |  |
| **Drinking frequency** | **Never** | Reference | | **0.0049** | Reference | | 0.19 | Reference | | **0.018** |
|  | **Always** | 1.8 (1.26,2.58) | **0.0013** |  | 1.92 (0.94,3.9) | 0.072 |  | 1.97 (1.23,3.16) | **0.0051** |  |
|  | **Sometimes** | 1.41 (0.8,2.49) | 0.23 |  | 1.46 (0.32,6.72) | 0.63 |  | 1.99 (1.01,3.92) | **0.047** |  |
| **Site of primary tumor** | **Pyriform fossa** | Reference | | 0.96 | Reference | | 0.71 | Reference | | 0.42 |
|  | **Postwall** | 0.93 (0.57,1.54) | 0.79 |  | 0.56 (0.14,2.32) | 0.42 |  | 0.64 (0.32,1.25) | 0.19 |  |
|  | **Post-cricoid** | 1.02 (0.54,1.93) | 0.95 |  | 1.11 (0.27,4.63) | 0.88 |  | 1.04 (0.38,2.82) | 0.94 |  |
| **Laterality of primary tumor** | **Unilateral** | Reference | | **0.0029** | Reference | | 0.77 | Reference | | **0.0039** |
|  | **Both** | 2.27 (1.32,3.88) |  |  | 0.81 (0.19,3.39) |  |  | 2.68 (1.37,5.24) |  |  |
| **Differential grade** | **Low** | Reference | | **<0.001** | Reference | | 0.29 | Reference | | **0.02** |
|  | **High** | 2.12 (1.35,3.34) | **0.0012** |  | 2.2 (0.81,5.97) | 0.12 |  | 2.02 (1.1,3.68) | **0.022** |  |
|  | **Medium** | 0.77 (0.5,1.17) | 0.21 |  | 1.13 (0.45,2.82) | 0.8 |  | 0.74 (0.41,1.32) | 0.31 |  |
| **T category** | **T1/2** | Reference | | **0.016** | Reference | | 0.35 | / | | |
|  | **T3** | 1.72 (1.15,2.59) | **0.0088** |  | 0.76 (0.43,1.35) |  |  |  |  |  |
|  | **T4** | 1.58 (1.12,2.24) | **0.0098** |  | **/** | | |  |  |  |
| **N category** | **N0** | Reference | | 0.12 | Reference | | 0.21 | Reference | | **0.012** |
|  | **N1** | 1.27 (0.66,2.44) | 0.47 |  | 1.48 (0.8,2.75) |  |  | 1.27 (0.66,2.44) | 0.47 |  |
|  | **N2** | 1.75 (1.04,2.92) | **0.034** |  | / | | | 2.15 (1.25,3.68) | **0.0053** |  |
|  | **N3** | 1.62 (0.9,2.92) | 0.11 |  |  |  |  | 2.16 (1.08,4.33) | 0.03 |  |
| **M category** | **M0** | Reference | | **<0.001** | / | | | Reference | | **<0.001** |
|  | **M1** | 3.02 (1.65,5.55) |  |  |  |  |  | 5.95 (2.73,12.93) | |  |
| **Surgery** | **No** | Reference | | **<0.001** | Reference | | **0.019** | Reference | | **<0.001** |
|  | **Yes** | 0.55 (0.41,0.73) |  |  | 0.48 (0.26,0.88) |  |  | 0.38 (0.26,0.55) |  |  |
| **Chemotherapy** | **No** | Reference | | 0.37 | Reference |  | 0.31 | Reference |  | **0.015** |
|  | **Yes** | 1.17 (0.83,1.63) |  |  | 0.73 (0.4,1.34) |  |  | 1.78 (1.12,2.84) |  |  |
| **Radiotherapy** | **No** | Reference | | 0.063 | Reference | | 0.35 | Reference | | 0.41 |
|  | **Yes** | 0.77 (0.58,1.01) |  |  | 0.73 (0.38,1.41) |  |  | 0.86 (0.6,1.23) |  |  |
| **Combined treatment modality** | **SRC** | Reference | | **<0.001** | Reference | | **0.061** | Reference | | **<0.001** |
|  | **chemotherapy alone** | 2.83 (1.8,4.45) | **<0.001** |  | 11.79 (2.24,61.97) | **0.0036** |  | 3.48 (1.96,6.18) | **<0.001** |  |
|  | **RT alone** | 3.04 (1.49,6.21) | **0.0023** |  | 12.47 (2.25,69.26) | **0.0039** |  | 3.32 (1.32,8.35) | **0.011** |  |
|  | **CRT** | 1.72 (1.15,2.58) | **0.0081** |  | 5.71 (1.34,24.34) | **0.018** |  | 2.06 (1.23,3.46) | **0.0063** |  |
|  | **surgery alone** | 1.29 (0.75,2.22) | 0.35 |  | 6.35 (1.26,31.9) | **0.025** |  | 0.89 (0.42,1.87) | 0.76 |  |
|  | **SC** | 1.12 (0.44,2.89) | 0.81 |  | 2.04 (0.18,22.68) | 0.56 |  | 1 (0.29,3.36) | 0.99 |  |
|  | **SR** | 1.09 (0.52,2.28) | 0.83 |  | 4.64 (0.93,23.11) | 0.061 |  | 0.7 (0.24,2.07) | 0.52 |  |

HPSCC: hypopharyngeal squamous cell carcinoma; HR: hazard ratio; CI: confidence interval; SRC: surgery+ radiotherapy+ chemotherapy; RT: radiotherapy; CRT: chemoradiotherapy; SC: surgery+ chemotherapy; SR: surgery+ radiotherapy.

Supplementary Table 5: Multivariable Cox proportional hazards regression analysis evaluating the predictors of overall survival in stage IV, stage III, and category T4 HPSCC

| **Covariate** | | **Stage IV** | | | **Stage III** | | | **Category T4** | | |
| --- | --- | --- | --- | --- | --- | --- | --- | --- | --- | --- |
|  |  | **HR(95%CI)** | **p-value** | **Global p-value** | **HR(95%CI)** | **p-value** | **Global p-value** | **HR(95%CI)** | **p-value** | **Global p-value** |
| **Drinking frequency** | **Never** | Reference |  | 0.4 | / | | | Reference |  | 0.59 |
|  | **Always** | 1.40 (0.77,2.54) | 0.27 |  |  |  |  | 1.19 (0.50,2.84) | 0.7 |  |
|  | **Sometimes** | 1.68 (0.75,3.72) | 0.2 |  |  |  |  | 1.70 (0.58,4.96) | 0.33 |  |
| **Laterality of primary tumor** | **Unilateral** | Reference |  | 0.11 | / | | | Reference |  | **0.03** |
|  | **Both** | 1.85 (0.87,3.94) |  |  |  |  |  | 3.07 (1.11,8.50) |  |  |
| **Differential grade** | **Low** | Reference |  | **0.033** | / | | | Reference |  | **0.042** |
|  | **High** | 2.51 (1.28,4.94) | **0.008** |  |  |  |  | 3.95 (1.47,10.63) | 0.007 |  |
|  | **Medium** | 0.85 (0.47,1.54) | 0.6 |  |  |  |  | 1.43 (0.62,3.31) | 0.41 |  |
| **T category** | **T1/2** | Reference |  | 0.79 | / | | | / | | |
|  | **T3** | 1.25 (0.64,2.44) | 0.52 |  |  |  |  |  |  |  |
|  | **T4** | 1.20 (0.67,2.15) | 0.55 |  |  |  |  |  |  |  |
| **N category** | **N0** | / | | | / | | | Reference |  | 0.6 |
|  | **N1** |  |  |  |  |  |  | 1.23 (0.36,4.14) | 0.74 |  |
|  | **N2** |  |  |  |  |  |  | 1.77 (0.63,4.96) | 0.27 |  |
|  | **N3** |  |  |  |  |  |  | 1.84 (0.37,9.07) | 0.45 |  |
| **M category** | **M0** | Reference |  | 0.87 | / | | | Reference |  | 0.61 |
|  | **M1** | 1.11 (0.33,3.76) |  |  |  |  |  | 0.59 (0.08,4.27) |  |  |
| **Surgery** | **No** | Reference |  | **0.002** | Reference |  | **<0.001** | Reference |  | **<0.001** |
|  | **Yes** | 0.44 (0.26,0.75) |  |  | 0.25 (0.11,0.55) |  |  | 0.30 (0.15,0.60) |  |  |
| **Radiation** | **No** | Reference |  | 0.72 | Reference |  | 0.15 | Reference |  | 0.95 |
|  | **Yes** | 0.91 (0.56,1.48) |  |  | 0.59 (0.29,1.20) |  |  | 0.98 (0.50,1.93) |  |  |
| **Chemotherapy** | **No** | Reference |  | 0.062 | Reference |  | **0.013** | Reference |  | 0.061 |
|  | **Yes** | 0.56 (0.31,1.03) |  |  | 0.39 (0.19,0.82) |  |  | 0.46 (0.20,1.04) |  |  |

HPSCC: hypopharyngeal squamous cell carcinoma; HR: hazard ratio; CI: confidence interval

Supplementary Figure 1: Patient inclusion and exclusion flowchart

HPSCCC: hypopharyngeal squamous cell carcinoma; WCH: West China Hospital; SCH: Sichuan Cancer Hospital.


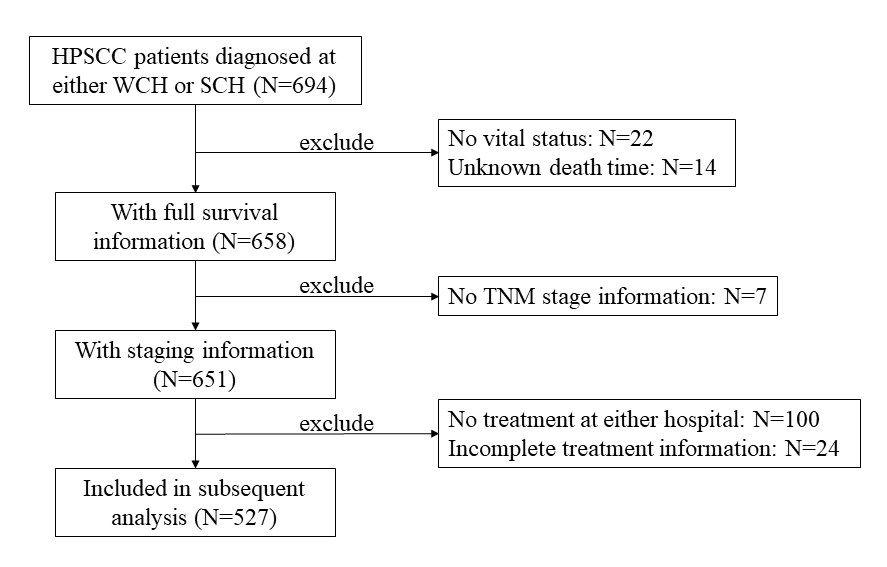


Supplementary Figure 2: Comparison of overall survival between HPSCC patients treated with RT alone and those treated with surgery/SBMT highlighted the effects of surgery

Note: HRs were derived from univariate Cox proportional hazards regression models. The p-values were generated using log-rank tests. HPSCC: hypopharyngeal squamous cell carcinoma; RT: radiotherapy; SBMT: surgery-based multimodality treatment; HR: hazards ratio.


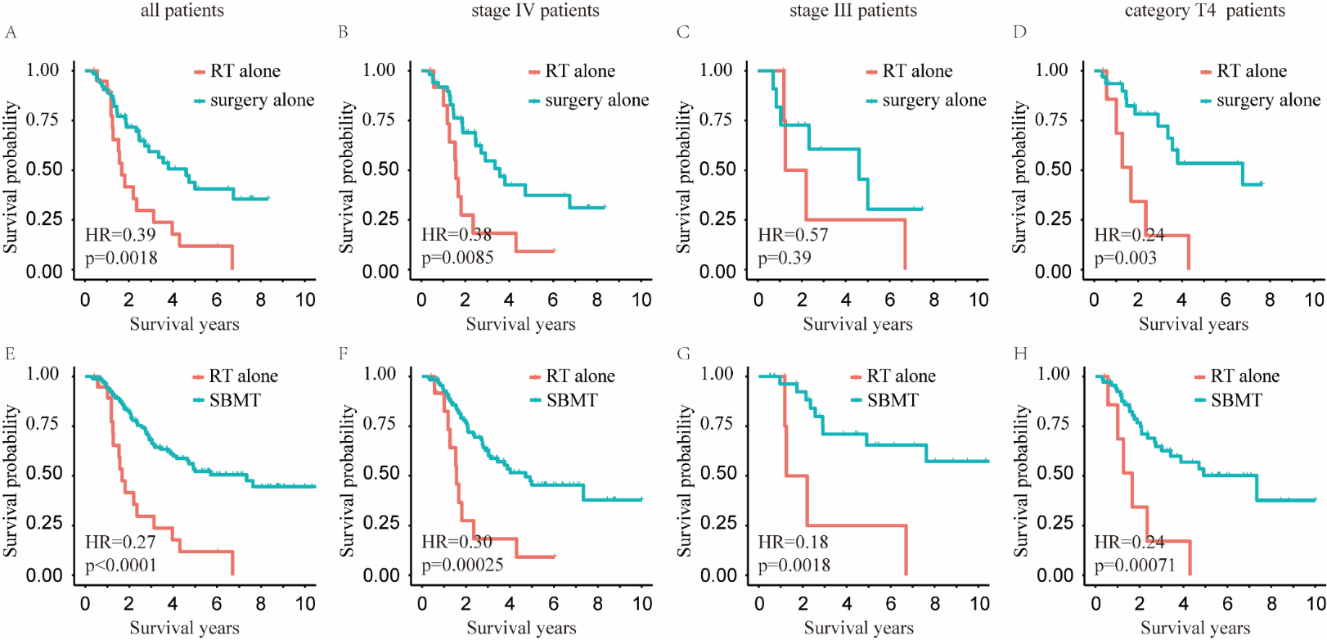


Supplementary Figure 3: Comparison of overall survival between HPSCC patients treated with RT alone and those treated with different types of SBMT

Note: HRs were derived from univariate Cox proportional hazards regression models. The p-values were generated using log-rank tests. HPSCC: hypopharyngeal squamous cell carcinoma; RT: radiotherapy; SBMT: surgery-based multimodality treatment; SC: surgery+ chemotherapy; SR: surgery+ radiotherapy; SRC: surgery+ radiotherapy + chemotherapy; HR: hazards ratio.

**
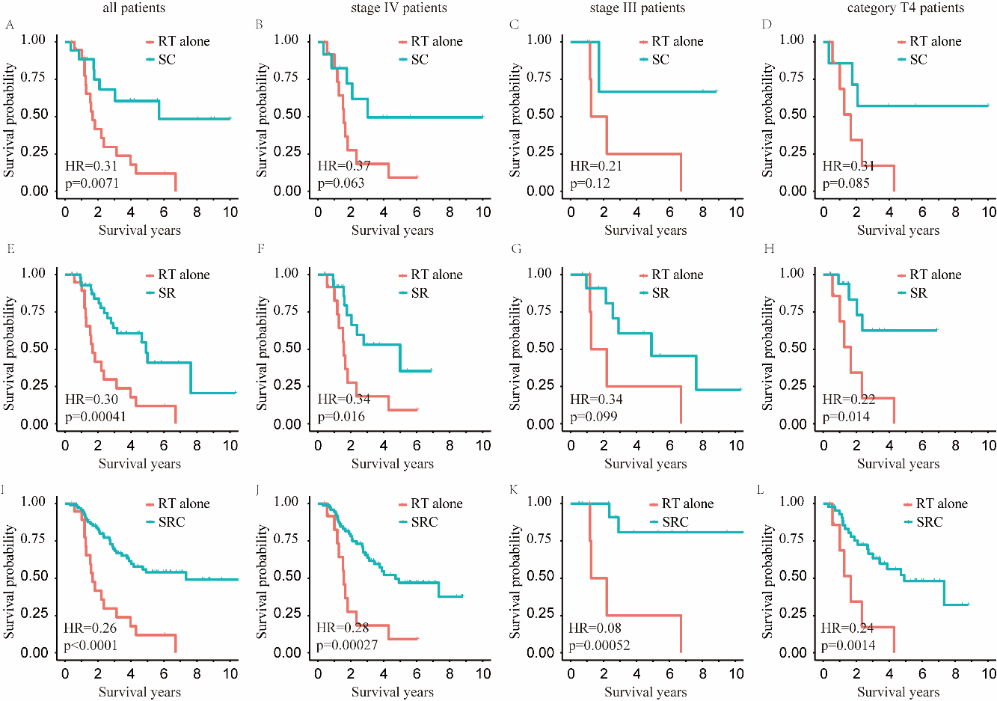
**

Supplementary Figure 4: Comparison of overall survival between HPSCC patients treated with CRT and those treated with different types of SBMT

Note: HRs were derived from univariate Cox proportional hazards regression models. The p-values were generated using log-rank tests. HPSCC: hypopharyngeal squamous cell carcinoma; CRT: chemoradiotherapy; SBMT: surgery-based multimodality treatment; SC: surgery+ chemotherapy; SR: surgery+ radiotherapy; SRC: surgery+ radiotherapy+ chemotherapy; HR: hazards ratio.


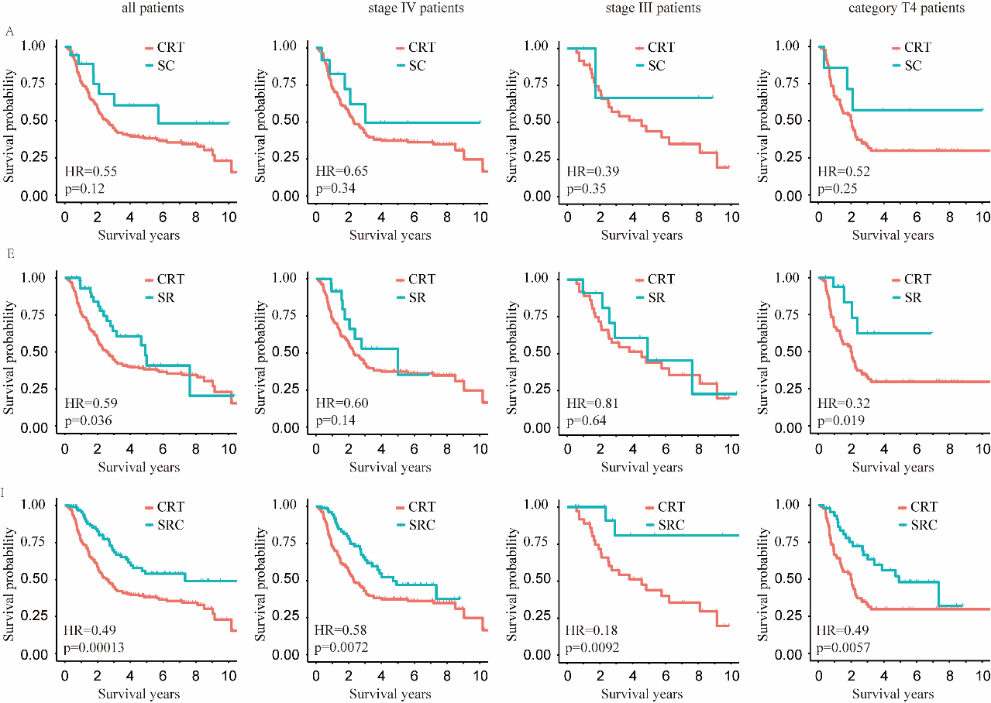


Supplementary Figure 5: Comparison of overall survival between HPSCC patients treated with surgery alone and SBMT, and between HPSCC patients treated with RT alone and CRT, respectively

Note: HRs were derived from univariate Cox proportional hazards regression models. The p-values were generated using log-rank tests. HPSCC: hypopharyngeal squamous cell carcinoma; SBMT: surgery-based multimodality treatment; RT: radiotherapy; CRT: chemoradiotherapy; HR: hazards ratio.


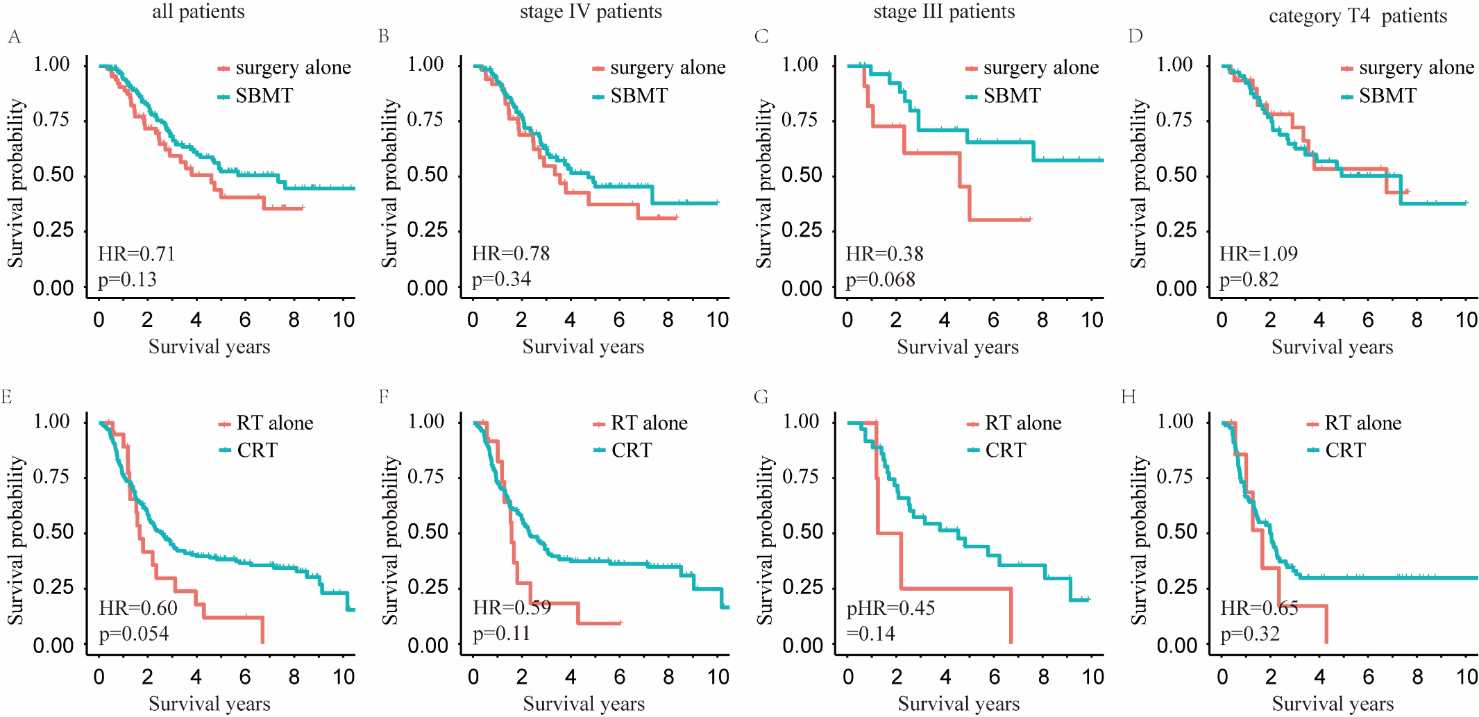


Supplementary Figure 6: Comparison of overall survival between HPSCC patients treated with surgery alone and those treated with different types of SBMT

Note: HRs were derived from univariate Cox proportional hazards regression models. The p-values were generated using log-rank tests. HPSCC: hypopharyngeal squamous cell carcinoma; SBMT: surgery-based multimodality treatment; SC: surgery+ chemotherapy; SR: surgery+ radiotherapy; SRC: surgery+ radiotherapy+ chemotherapy; HR: hazards ratio.


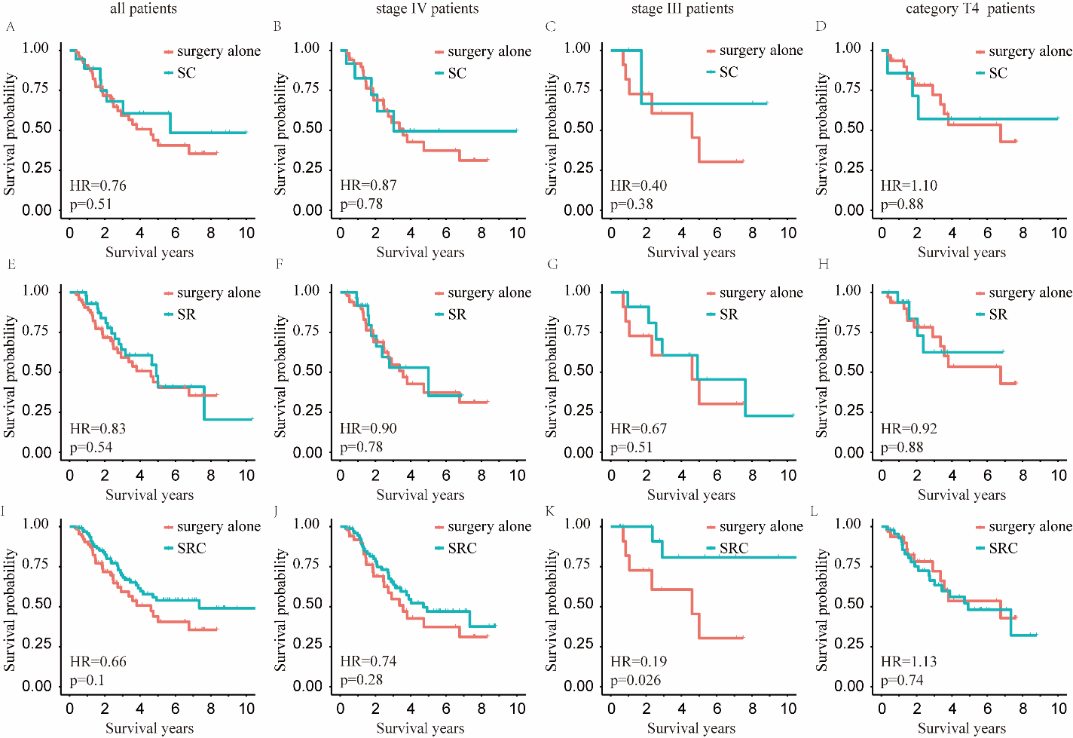

Supplement: Multimedia component 1 [file mmc1.docx]
